# Injury Burden in India, 1990 to 2023: National and Subnational Trends From the Global Burden of Disease 2023 Study

**DOI:** 10.7759/cureus.115452

**Published:** 2026-08-30

**Authors:** Rajan Kaushik, Gaurav Vashistha

**Affiliations:** 1 Community Medicine, Adesh Medical College and Hospital, Kurukshetra, IND; 2 Pediatric Medicine, Adesh Medical College and Hospital, Kurukshetra, IND

**Keywords:** disability-adjusted life years, ecological analyses, gbd 2023, india, injury burden, temporal trend analysis

## Abstract

Background: In India, injuries are considered a public health issue since they cause a great deal of death, disability and pressure on the healthcare system; yet there has been no thorough evaluation of the national and subnational temporal trends in the burden of injury using the data from the Global Burden of Disease (GBD) estimates.

Objectives: To assess temporal trends in the aggregate burden of all injuries, including incidence, prevalence, mortality, and disability-adjusted life years (DALYs), in India and its states and union territories from 1990 to 2023, and to describe variations in injury burden by age and sex. Temporal trends were evaluated using estimated annual percentage change (EAPC) and Joinpoint regression.

Methods: We conducted an ecological time-trend study using GBD 2023 estimates for India and its states and union territories. Age-standardized incidence, prevalence, death and DALY rates were analysed from 1990 to 2023. Temporal trends were examined using EAPC and Joinpoint regression analysis. State-level differences, changes in rankings, and age- and sex-specific patterns were also examined. A burden-trend quadrant analysis was used to identify states with high burden and slower or faster improvement.

Results: Between 1990 and 2023, incident cases increased from 100.99 million to 126.36 million; prevalent cases increased from 163.01 million to 271.61 million; deaths increased from 660,695 to 953,809; and DALYs increased from 44.38 million to 52.51 million. On the other hand, age-standardized incidence, prevalence, death and DALY rates declined by 26.8%, 20.5%, 27.5% and 34.1%, respectively. Joinpoint analysis showed significant overall declines in age-standardized incidence rate (ASIR) (average annual percent change (AAPC) -0.92%), age-standardized prevalence rate (ASPR) (-0.67%), age-standardized death rate (ASDR) (-0.89%) and age-standardized DALY rate (ASDAR) (-1.15%) (all P<0.001). Considerable interstate variation persisted, with Telangana, Uttarakhand and Chhattisgarh being the states with the highest DALY rates in 2023. Males had higher mortality and DALY rates than females, while mortality and DALY rates increased markedly with increasing age.

Conclusion: India faced a decline in age-standardized injury burden from 1990 to 2023 despite increasing absolute numbers. Persistent geographic, sex and age-related differences point toward the need for specific injury prevention, strengthened surveillance and state-specific public health strategies.

## Introduction

Injuries are a major public health concern globally and are defined as acute exposures to agents such as heat, electricity, mechanical energy, chemicals, or radiation that exceed the human tolerance limit and also include the sudden absence of essential agents like oxygen or heat. Based on intention, injuries are classified as unintentional, intentional, or of undetermined intent, covering a large variety of conditions that play a major role in mortality, morbidity, disability, and economic loss [1].

Injuries cause many deaths and disability around the world. In 2019, injuries were responsible for nearly 4.4 million deaths, making up nearly 8% of all deaths globally, and were also responsible for about 8% of all years lived with disability (YLDs). Among younger individuals (5-29 years old), roadside injuries, homicide, and suicide were three of the five top causes of death. Falls alone contributed more than 684,000 deaths globally in 2019, while millions more people suffer non-fatal injuries that lead to emergency care visits, hospitalizations, disability and the need for long-term rehabilitation and healthcare services [2].

India holds a large share of the global burden of injury. In 2017, injuries were responsible for nearly 9.5 lakh deaths and 5.53 crore DALYs. The largest burden occurred among adults aged 15-59 years, responsible for nearly 5.9 lakh deaths and 3.91 crore DALYs, which shows economic losses during the years of life with working potential. Males experienced a higher burden than females, while accidental injuries, falls, intentional self-harm, drowning, and venomous animal encounters remained among the top contributors to injury-related DALYs throughout various age groups [3].

In addition to health consequences, injuries also cause a financial burden on society and health systems. Due to accidents and injuries, 264.7 out of 10,000 persons need hospitalization per year, with an average hospital stay of 8.77 days in India. Out-of-pocket expenditure (OOPE) for care of injured persons in private health facilities is nearly five times higher than in public facilities, with mean OOPE accounting for approximately 97% and 91% of total health expenditure in public and private health facilities, respectively. The economic burden is higher among the elderly, males, high socio-economic households, individuals with higher educational qualifications, and those availing treatment in private hospitals, whereas health insurance has been shown to reduce OOPE [4].

The Global Burden of Disease (GBD) 2023 study was conducted by the Institute for Health Metrics and Evaluation (IHME) and gives detailed, uniform measures of disease burden across several geographical locations, including countries, territories, age groups, sexes, and time periods [6]. According to GBD 2023, data were collected from different sources, including vital registration systems, hospital records, disease registries, household surveys, surveillance systems and published literature. Advanced statistical models, like Bayesian meta-regression (DisMod-MR 2.1), Cause of Death Ensemble model (CODEm), and spatiotemporal Gaussian process regression (ST-GPR) were used to generate consistent estimates of disease burden while taking into account for incomplete and heterogeneous data. The study shows disease burden as incidence, prevalence, mortality, years of life lost (YLLs), YLDs, and DALYs, each presented with 95% uncertainty intervals [5]. Within GBD 2023, injuries are further classified by cause (e.g., road injuries, falls, interpersonal violence) and nature (e.g., fractures or traumatic brain injury), supporting detailed assessment of fatal and non-fatal injury outcomes in future research [6].

Recent global analyses based on GBD data show that injury incidence, prevalence, deaths, and DALYs have increased in absolute numbers since 1990 due to population growth and ageing, but age-standardized rates (ASRs) have declined. But differences remain across geographic regions, sexes, and levels of socio-demographic development, with males and populations in low Socio-Demographic Index (SDI) regions consistently experiencing higher injury burdens [7].

Despite the availability of standardized estimates from GBD 2023, studies that comprehensively examine the burden of injuries across India and its states using these estimates remain limited. Limited studies describe trends across time periods and demographic variations in injury burden by age and sex. Addressing these gaps is essential to identify high-risk populations, understand geographical disparities, and inform research-based strategies for injury prevention and health policy.

Therefore, this study aims to assess the aggregate burden of all injuries in India and its states and union territories from 1990 to 2023 using data from the GBD 2023 database. Specifically, the study aims to describe the incidence, prevalence, mortality, and DALYs attributable to all injuries and to examine variations in injury burden across age groups, sex, and geographical locations. The study further aims to assess temporal trends in these injury burden measures using estimated annual percentage change (EAPC) and Joinpoint regression.

## Materials and methods

Study design

Secondary data-based descriptive ecological time-trend study examining injury burden trends at national and subnational (State) levels in India between 1990 and 2023 from GBD Study 2023 data. It tracked changes over 34 years in injury incidence, prevalence, mortality, and DALYs. The study followed the Guidelines for Accurate and Transparent Health Estimates Reporting (GATHER) checklist [8].

Data source

Data were obtained from the GBD Study 2023, accessed through the Global Health Data Exchange (GHDx), managed by the IHME at the University of Washington in Seattle, USA [9]. The GBD uses standardized methods to estimate disease burden for multiple geographic regions, drawing from sources like civil registration, vital statistics, censuses, surveys, disease registries, hospital records, surveillance systems, and published research. When data are missing or incomplete, validated statistical modeling is used to make estimates comparable across locations, age groups, sexes, and years [10].

The present analysis focused on the aggregate “Injuries” cause category in the GBD cause hierarchy. Data were extracted for India and the 31 GBD geographical units representing Indian states and union territories, including the combined Jammu & Kashmir and Ladakh category and the combined “Other Union Territories” category, for each year from 1990 to 2023. The GBD Results Tool was used to extract estimates for incidence, prevalence, deaths, and DALYs. For the primary national and subnational trend analyses, ASRs and corresponding 95% uncertainty intervals (UIs) were extracted. Sex-specific estimates were obtained for males, females, and both sexes, while age-specific estimates were obtained for the selected GBD-defined age groups. The data extraction was performed on 27th July 2026. The exact GBD Results Tool extraction parameters are provided in the Appendix to facilitate reproducibility.

Outcome measures

The main outcomes were the age-standardized incidence rate (ASIR), age-standardized prevalence rate (ASPR), age-standardized death rate (ASDR), and age-standardized DALY rate (ASDAR). ASRs were used to account for variation in population age composition and to allow comparisons between states and over different time periods. The GBD study uses the world standard population of GBD for age standardization [11]. Secondary outcomes included state-specific burden, sex and age differences, changes in disease burden over time, and shifts in state rankings from 1990 to 2023.

Data management

Data were obtained from the GBD Results Tool and imported into R version 4.6.1 (R Foundation for Statistical Computing, Vienna, Austria) for preprocessing and statistical analysis. The data were filtered to include India and all its states and union territories, focusing on ASRs for the main analysis. Separate datasets were prepared for national trends, state comparisons, sex, age, and Joinpoint regression. Before analysis, datasets were checked for duplicates, missing data, coding inconsistencies, and completeness to ensure quality and reproducibility. 

Statistical analysis

The analysis began by summarizing the epidemiological impact of injuries in India. National trends for ASIR, ASPR, ASDAR, and ASDR from 1990 to 2023 were shown with line graphs, while choropleth maps illustrated geographic variation. State rankings based on age-standardized DALY rates were created for 1990 and 2023. Absolute and percentage changes during the study period were calculated to measure changes in injury burden among states. The long-term temporal trends were determined by means of the EAPC for each outcome measure; a log-linear regression model was utilized using time in calendar year as the independent variable and the natural logarithm of the ASR as the dependent variable [12,13].

$\ln(ASR)=\alpha+\beta(Year)+\epsilon$

where α denotes the intercept, β represents the annual change (slope) in the natural logarithm of the ASR, and ε is the random error term. The EAPC was calculated as:

$EAPC=100\times (e^{\beta}-1)$

Positive EAPC shows an increasing trend, while negative indicates a decreasing trend. Confidence intervals (95% CIs) were calculated for all estimates. To detect changes in temporal trends, we carried out a joinpoint regression analysis using the Joinpoint Regression Program version 6.1.0 (Statistical Methodology and Applications Branch, National Cancer Institute, Bethesda, MD, USA) [14]. The standard errors (SE) needed for the Joinpoint modelling were calculated from the given 95% uncertainty intervals using the following formula:

$SE=\frac{Upper UI-Lower UI}{2\times 1.96}$

where Upper UI represents the upper limit of the 95% GBD uncertainty interval and Lower UI represents the lower limit of the 95% GBD uncertainty interval and assuming an approximately symmetric normal distribution on the scale used for the regression. This approach treats the reported GBD uncertainty intervals as an approximation for deriving the variability required by the Joinpoint procedure and does not imply that GBD uncertainty intervals are conventional sampling-based confidence intervals

The model chosen was the Weighted Bayesian Information Criterion (BIC), as there were 34 annual observations and up to three joinpoints allowed [15]. The Annual Percent Change (APC) was calculated for all trend segments, and the Average Annual Percent Change (AAPC) showed the trend for the time period. Statistical significance was assessed using two-sided tests, with a p-value below 0.05 taken as significant. Annual GBD estimates may exhibit temporal dependence because estimates across adjacent years are generated within a common modelling framework and may share underlying data sources and modelling assumptions. The study did not have access to the year-specific variance-covariance matrix and therefore did not explicitly model temporal covariance between annual GBD estimates. Consequently, the statistical significance of Joinpoint trends should be interpreted as an analytical approximation based on the available annual estimates and their derived standard errors.

State-level changes in injury burden were examined using state transition analysis. To prioritize public health issues, a burden-trend quadrant analysis was used. States and union territories were classified according to their ASDAR in 2023, representing the current injury burden, and their EAPC in DALY rate from 1990 to 2023, representing the temporal trend. The median ASDAR across the included GBD geographical units was used as the burden cut-off, while the median EAPC was used as the trend cut-off. States with ASDAR greater than or equal to the median and EAPC greater than or equal to the median were classified as high burden with slow improvement. States with ASDAR greater than or equal to the median and EAPC below the median were classified as high burden with rapid improvement. States with ASDAR below the median and EAPC greater than or equal to the median were classified as low burden with slow improvement, while those with ASDAR below the median and EAPC below the median were classified as low burden with rapid improvement. Thus, observations exactly equal to either median cut-off were classified as high burden or slow improvement, respectively. The median values were 3427 per 100,000 population for ASDAR and -1.17% FOR EAPC in DALY rates, respectively. This approach helped assess both the current disease burden and the rate of change, making it easier to identify areas needing stronger injury prevention strategies.

Separate analyses were done for different sexes (Men and Women) to examine variation in injury burden and trends over time. Age-based analysis covered selected groups defined in the GBD to see how injury burden varies across life stages. Spatial variation among states and union territories was shown with choropleth maps based on state boundary shapefiles. ASRs were grouped using the Fisher-Jenks natural breaks algorithm, which reduces variance within each class and highlights geographic differences. The analysis and visualisations were carried out using R version 4.6.1 and the following packages: tidyverse, ggplot2, sf, ggrepel, patchwork, viridis, RColorBrewer, openxlsx, janitor, and classInt. The joinpoint analyses were completed with the Joinpoint Regression Program version 6.1.0. The analytical scripts were kept under version control in order to ensure reproducibility.

Ethical considerations

This study used only anonymised secondary data from the GBD 2023 study available publicly and did not include any direct involvement with human subjects or access to any identifiable personal information; as a result, it was not necessary to obtain approval from an Institutional Ethics Committee or to secure informed consent in line with both institutional and national guidelines.

Data availability

The datasets analysed in this study can be obtained from the GBD 2023 study via the GHDx. Using the GBD Results Tool, it is possible to access all the estimates employed in this study, such as the age-standardized incidence, prevalence, mortality and DALY rates for India and its states and union territories. The analytical datasets produced during the study and the R scripts used for data preprocessing, analysis, and figure generation are available from the corresponding author upon reasonable request.

## Results

Temporal trends of injuries

From 1990 to 2023 the total impact of injuries in India increases considerably according to all the different outcome indicators: the number of new cases increases from 100.99 million to 126.36 million (a 25.1% increase), the number of existing cases increased from 163.01 million to 271.61 million (a 66.6% increase), the number of deaths went from 660,695 to 953,809 (an increase of 44.4%), and DALYs moved from 44.38 million to 52.51 million (an increase of 18.3%) (Table 1).

**Table 1:** National burden of injuries in India, 1990 and 2023 (counts, age-standardized rates, and percentage change) Age-standardized rates are expressed per 100,000 population. Estimates were obtained from the Global Burden of Disease (GBD) Study 2023 [9]. Abbreviations: ASR: Age-standardized rate, DALYs: Disability Adjusted Life Years

Measure	Metric	Year 1990	Year 2023	Percent change (%)
Incidence	Number	100,988,950 (91,991,146-112,737,030)	126,359,326 (115,796,691-138,464,797)	25.1
	ASR (per 100,000)	11,717.50 (10,754.33-12,885.18)	8,577.59 (7,879.21-9,386.87)	-26.8
Prevalence	Number	163,006,461 (153,017,726-175,142,182)	271,614,041 (254,564,488-291,613,535)	66.6
	ASR (per 100,000)	23,738.62 (22,366.08-25,471.40)	18,878.68 (17,737.61-20,214.46)	-20.5
DALYs	Number	44,379,227 (37,418,896-50,065,051)	52,506,894 (44,089,974-60,005,932)	18.3
	ASR (per 100,000)	5,544.26 (4,716.15-6,242.07)	3,652.29 (3,067.26-4,165.76)	-34.1
Deaths	Number	660,695 (534,708-750,440)	953,809 (773,531-1,098,502)	44.4
	ASR (per 100,000)	100.39 (81.34-114.35)	72.81 (59.98-84.06)	-27.5

Despite growth in the absolute burden, each age-standardized injury indicator showed a reduction over the study period. The ASIR dropped by 26.8%, the ASPR by 20.5%, the ASDR by 27.5%, and the ASDAR by 34.1% between 1990 and 2023 (Table 1, Figure 1).

**Figure 1:**
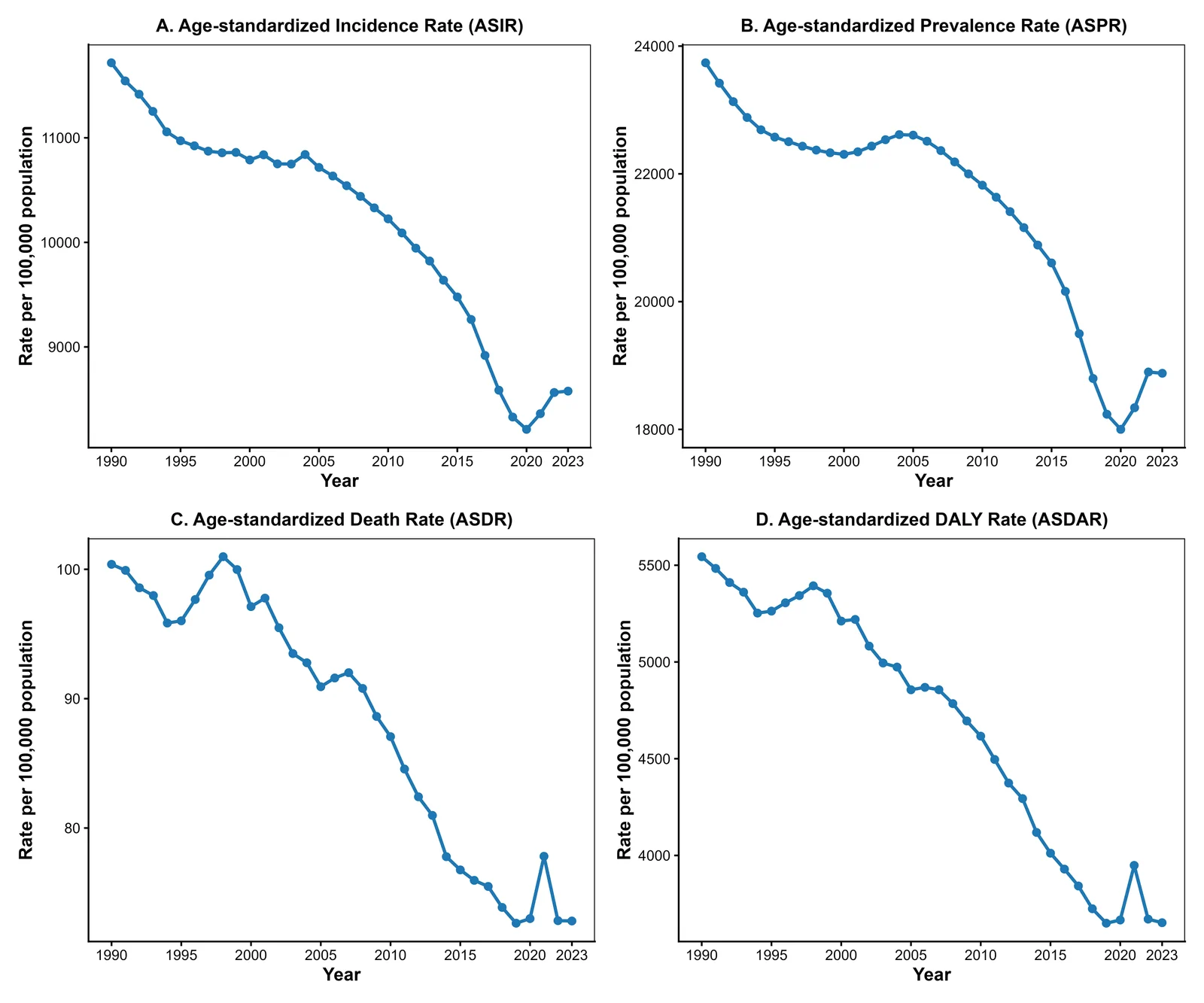
National trends in age-standardized (A) Incidence, (B) Prevalence, (C) Death, and (D) Disability-adjusted life year (DALY) rates for injuries in India, 1990-2023. Age-standardized rates are expressed per 100,000 population. Estimates were obtained from the Global Burden of Disease (GBD) Study 2023 [9].

Geographic variation in injury burden across Indian states

Geographic variation in the burden of injury among the states and union territories of India in 2023 is shown in Figures 2, 3. Telangana had the highest ASDAR at 5,403.0 per 100,000 population, then came Uttarakhand at 4,815.6 and Chhattisgarh at 4,547.2, while Delhi had the lowest at 2,064.5, Mizoram at 2,069.7 and Kerala at 2,504.8. The same geographic pattern was seen with regard to ASDR; Telangana and Uttarakhand showed the highest mortality rates and Delhi and Mizoram the lowest.

**Figure 2:**
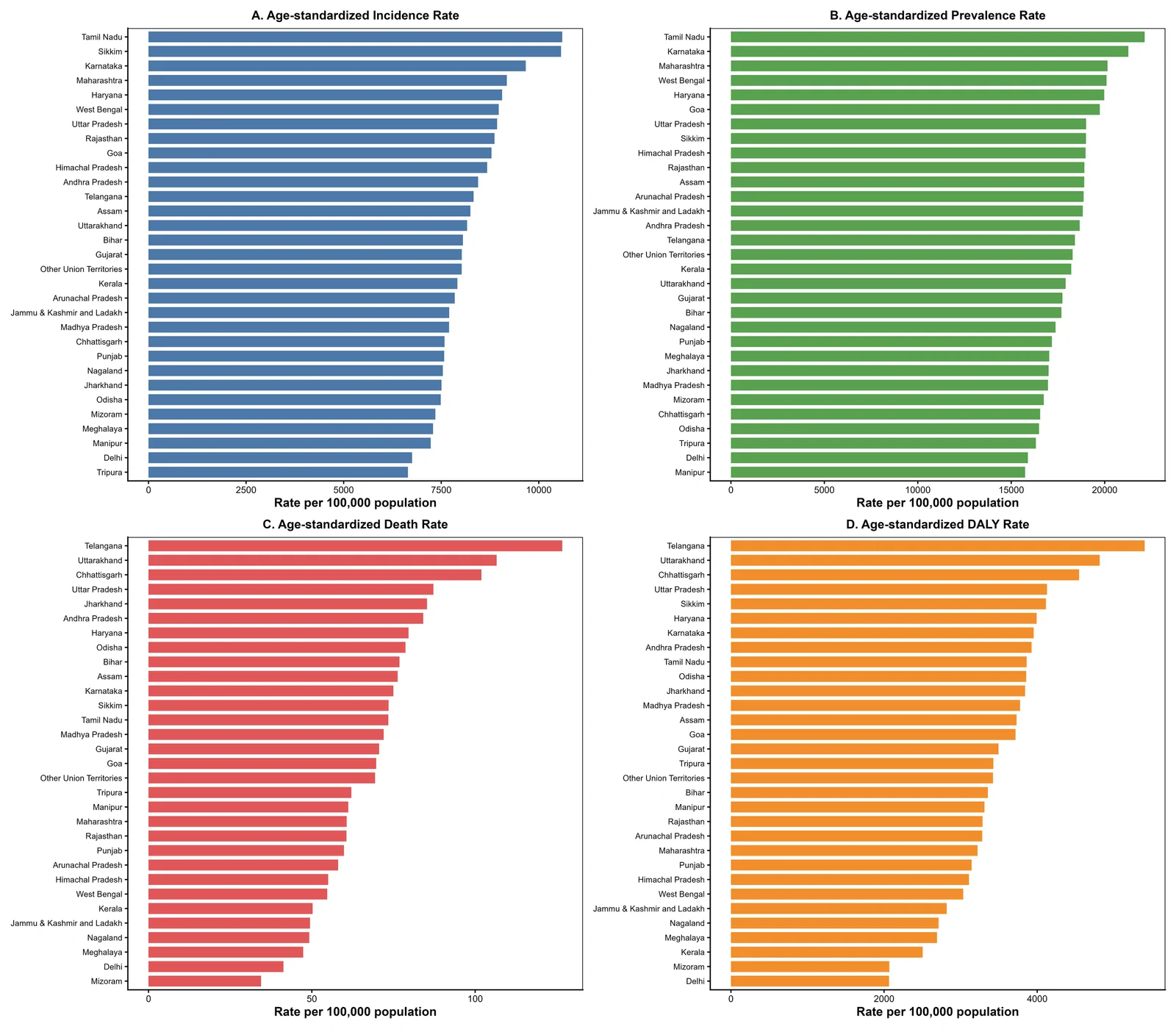
State-wise ranking of age-standardized (A) Incidence rates (ASIR), (B) Prevalence rates (ASPR), (C) Death rates (ASDR), and (D) Disability-adjusted life year (DALY) rates (ASDAR) for injuries across Indian states and Union territories in 2023. Estimates are presented for the administrative units defined by the Global Burden of Disease (GBD) Study 2023 [9], which may differ slightly from the current official administrative classification of India. In the GBD database, some recently created states or union territories are combined with their parent states or grouped as "Other Union Territories", and Jammu & Kashmir and Ladakh are reported together as a single unit. Consequently, the number and nomenclature of states and union territories shown in this figure may not exactly correspond to the present administrative divisions of India. Age-standardized rates are expressed per 100,000 population.

**Figure 3:**
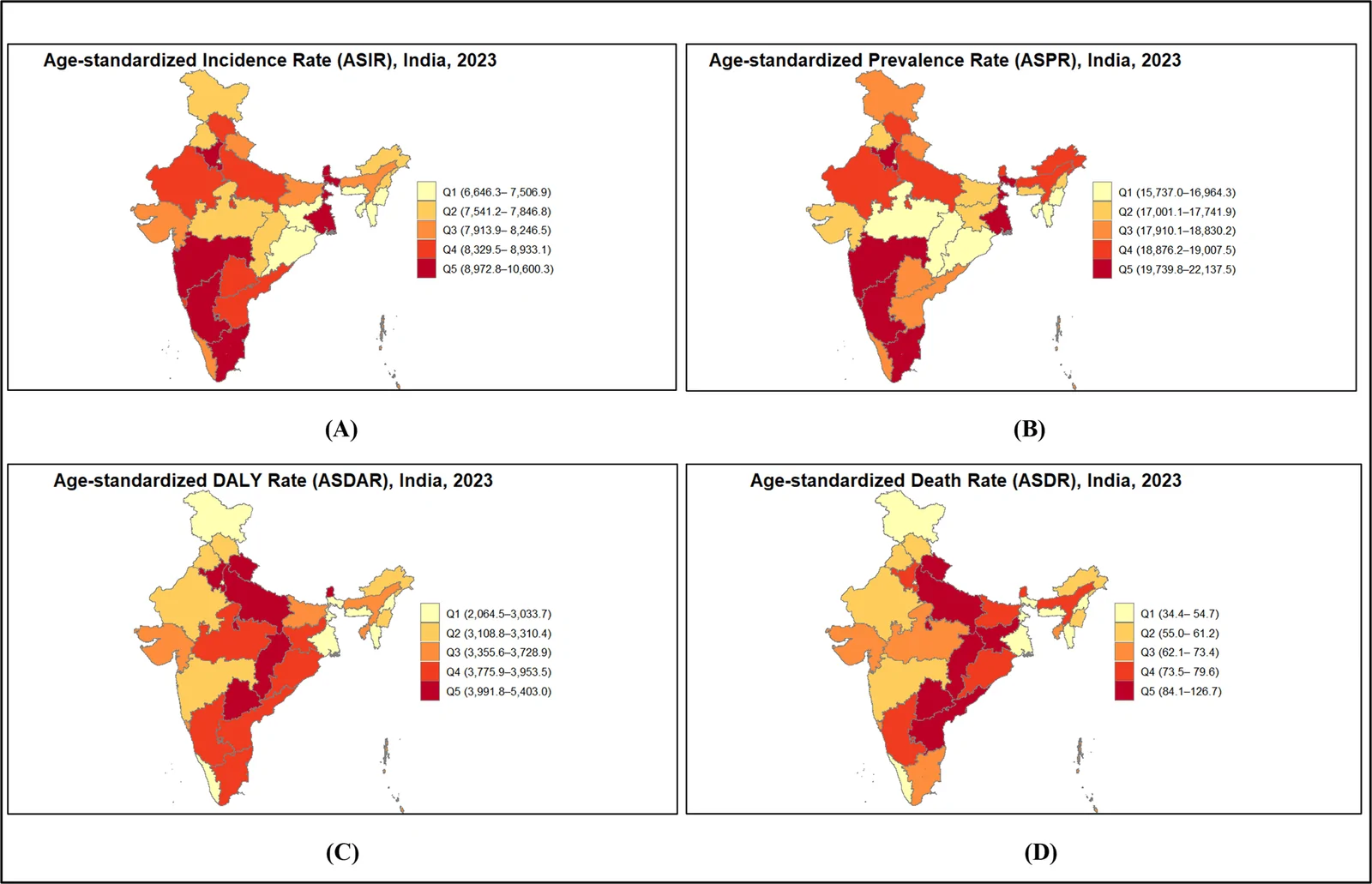
Geographic distribution of (A) age-standardized Incidence rates (ASIR), (B) Prevalence rates (ASPR), (C) Disability-adjusted life years (DALY) rates (ASDAR) and (D) Death rates (ASDR) for injuries across Indian states and union territories in 2023. Age-standardized rates are expressed per 100,000 population. Estimates were obtained from the Global Burden of Disease (GBD) Study 2023 [9]. States and Union territories were classified into five Fisher-Jenks (natural break) classes based on the distribution of each age-standardized rate. For simplicity of presentation, the classes are labelled Q1-Q5, where Q1 represents the lowest burden category and Q5 represents the highest burden category. Darker shades indicate higher rates. This figure was created by the authors using R software (R Foundation for Statistical Computing, Vienna, Austria), utilizing open-access boundary shapefiles provided by the Survey of India (surveyofindia.gov.in).

Long-term temporal trends across states

A downward trajectory in ASIRs was observed across all states, although the magnitude of change varied considerably (see Table 2). Nationally, the EAPC was -0.98% for ASIR, -0.71% for ASPR, -1.11% for ASDR, and -1.38% for ASDAR. Tamil Nadu had the steepest annual decrease in ASDAR (-2.05% per year), then came West Bengal (-2.04%) and Jammu & Kashmir and Ladakh (-1.98%). On the other hand, Arunachal Pradesh (-0.46%) and Uttarakhand (-0.60%) displayed the smallest reductions in ASDAR. Similar patterns were seen regarding mortality, incidence, and prevalence, even though the annual declines were generally higher for DALYs and deaths than for incidence and prevalence (Table 2).

**Table 2:** Estimated annual percentage change (EAPC) in age-standardized rates by state, 1990–2023. Estimates are presented for the administrative units defined by the Global Burden of Disease (GBD) Study 2023 [9], which may differ slightly from the current official administrative classification of India. In the GBD database, some recently created states or union territories are combined with their parent states or grouped as "Other Union Territories", and Jammu & Kashmir and Ladakh are reported together as a single unit. Consequently, the number and nomenclature of states and union territories shown in this figure may not exactly correspond to the present administrative divisions of India. Age-standardized rates are expressed per 100,000 population.

State	Incidence	Prevalence	DALYs	Deaths
Andhra Pradesh	-1.17 (-1.29 to -1.06)	-0.84 (-0.95 to -0.73)	-1.55 (-1.72 to -1.39)	-1.43 (-1.65 to -1.21)
Arunachal Pradesh	-0.43 (-0.85 to -0.00)	-0.54 (-0.66 to -0.42)	-0.46 (-1.13 to 0.22)	-0.29 (-1.14 to 0.57)
Assam	-0.96 (-1.10 to -0.82)	-0.63 (-0.75 to -0.51)	-1.67 (-1.84 to -1.49)	-1.13 (-1.33 to -0.93)
Bihar	-1.04 (-1.17 to -0.90)	-0.64 (-0.76 to -0.51)	-1.81 (-1.93 to -1.68)	-1.06 (-1.23 to -0.89)
Chhattisgarh	-0.96 (-1.06 to -0.85)	-0.74 (-0.84 to -0.64)	-1.02 (-1.12 to -0.93)	-0.69 (-0.82 to -0.56)
Delhi	-1.07 (-1.21 to -0.93)	-0.85 (-0.97 to -0.72)	-1.83 (-2.12 to -1.53)	-1.68 (-1.98 to -1.38)
Goa	-0.47 (-0.62 to -0.32)	-0.32 (-0.46 to -0.17)	-0.93 (-1.08 to -0.79)	-1.07 (-1.26 to -0.88)
Gujarat	-0.72 (-0.89 to -0.55)	-0.43 (-0.57 to -0.30)	-0.81 (-1.17 to -0.44)	-0.56 (-0.94 to -0.18)
Haryana	-0.88 (-1.00 to -0.76)	-0.71 (-0.82 to -0.59)	-0.95 (-1.06 to -0.84)	-0.35 (-0.50 to -0.20)
Himachal Pradesh	-0.59 (-0.69 to -0.48)	-0.35 (-0.46 to -0.23)	-1.42 (-1.70 to -1.15)	-1.65 (-1.95 to -1.36)
India	-0.98 (-1.11 to -0.86)	-0.71 (-0.83 to -0.58)	-1.38 (-1.51 to -1.24)	-1.11 (-1.24 to -0.98)
Jammu & Kashmir and Ladakh	-1.38 (-1.68 to -1.09)	-0.41 (-0.56 to -0.27)	-1.98 (-2.31 to -1.65)	-2.13 (-2.45 to -1.80)
Jharkhand	-0.96 (-1.08 to -0.84)	-0.63 (-0.73 to -0.52)	-1.09 (-1.24 to -0.94)	-0.67 (-0.87 to -0.47)
Karnataka	-1.05 (-1.18 to -0.91)	-0.82 (-0.95 to -0.68)	-1.20 (-1.33 to -1.06)	-0.92 (-1.05 to -0.78)
Kerala	-0.44 (-0.66 to -0.23)	-0.26 (-0.46 to -0.07)	-1.45 (-1.69 to -1.20)	-1.46 (-1.73 to -1.20)
Madhya Pradesh	-0.98 (-1.09 to -0.87)	-0.71 (-0.81 to -0.60)	-1.46 (-1.63 to -1.30)	-1.37 (-1.53 to -1.21)
Maharashtra	-0.72 (-0.84 to -0.61)	-0.51 (-0.63 to -0.39)	-1.44 (-1.59 to -1.29)	-1.49 (-1.66 to -1.32)
Manipur	-0.97 (-1.24 to -0.71)	-0.51 (-0.65 to -0.37)	-1.21 (-1.45 to -0.97)	-1.07 (-1.32 to -0.82)
Meghalaya	-0.71 (-0.90 to -0.52)	-0.52 (-0.68 to -0.36)	-0.80 (-0.98 to -0.62)	-0.74 (-0.96 to -0.52)
Mizoram	-1.02 (-1.27 to -0.77)	-0.74 (-0.95 to -0.52)	-1.62 (-1.77 to -1.46)	-1.67 (-1.88 to -1.47)
Nagaland	-0.63 (-0.78 to -0.48)	-0.40 (-0.54 to -0.26)	-1.23 (-1.40 to -1.06)	-1.08 (-1.29 to -0.88)
Odisha	-1.09 (-1.22 to -0.97)	-0.77 (-0.90 to -0.65)	-1.03 (-1.29 to -0.77)	-0.59 (-0.83 to -0.34)
Other Union Territories	-0.80 (-0.98 to -0.62)	-0.60 (-0.76 to -0.44)	-1.08 (-1.17 to -0.98)	-0.99 (-1.11 to -0.88)
Punjab	-0.85 (-1.01 to -0.70)	-0.52 (-0.66 to -0.38)	-1.17 (-1.33 to -1.01)	-0.98 (-1.14 to -0.82)
Rajasthan	-0.81 (-0.90 to -0.72)	-0.64 (-0.74 to -0.53)	-0.99 (-1.10 to -0.88)	-0.88 (-0.98 to -0.78)
Sikkim	-0.47 (-0.69 to -0.26)	-0.38 (-0.54 to -0.22)	-1.12 (-1.50 to -0.74)	-1.17 (-1.57 to -0.76)
Tamil Nadu	-1.35 (-1.48 to -1.22)	-1.07 (-1.19 to -0.95)	-2.05 (-2.31 to -1.79)	-2.02 (-2.33 to -1.71)
Telangana	-0.69 (-0.79 to -0.60)	-0.43 (-0.53 to -0.33)	-1.01 (-1.12 to -0.90)	-1.03 (-1.18 to -0.87)
Tripura	-0.95 (-1.14 to -0.76)	-0.51 (-0.66 to -0.36)	-1.11 (-1.33 to -0.87)	-1.05 (-1.29 to -0.82)
Uttar Pradesh	-1.00 (-1.15 to -0.86)	-0.83 (-0.98 to -0.68)	-0.99 (-1.12 to -0.85)	-0.31 (-0.40 to -0.22)
Uttarakhand	-0.72 (-0.82 to -0.62)	-0.44 (-0.53 to -0.35)	-0.60 (-0.76 to -0.45)	-0.58 (-0.75 to -0.41)
West Bengal	-1.06 (-1.21 to -0.92)	-0.76 (-0.90 to -0.63)	-2.04 (-2.18 to -1.90)	-1.88 (-2.04 to -1.73)

State transition and burden-trend prioritization

The burden-trend quadrant analysis showed that Telangana, Uttarakhand, Chhattisgarh, Haryana and Odisha were the states having a high current level of burden and relatively slow improvements; Tamil Nadu, Karnataka, Madhya Pradesh and Andhra Pradesh were those with a high burden but with comparatively rapid declines, while Delhi, Kerala, West Bengal, Jammu & Kashmir and Ladakh, and Mizoram were regarded as having a low burden together with rapid improvements. Meghalaya, Rajasthan, Punjab and Arunachal Pradesh were classified as having a low burden but with slower rates of improvement (see Figure 4).

**Figure 4:**
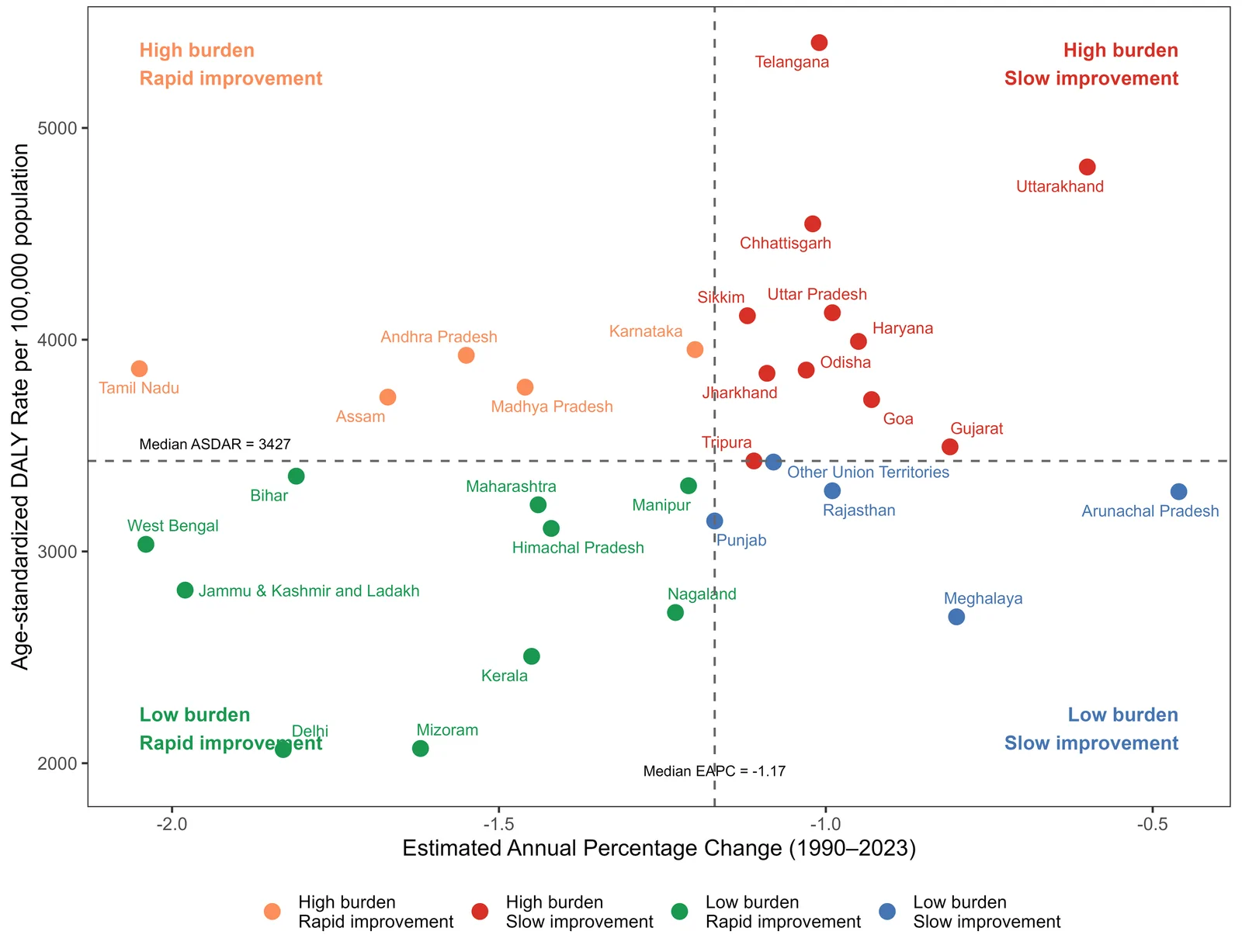
State-wise burden-trend quadrant analysis of injury burden in India based on age-standardized DALY rates in 2023 and estimated annual percentage change (EAPC), 1990-2023. Age-standardized rates are expressed per 100,000 population. Estimates were obtained from the Global Burden of Disease (GBD) Study 2023 [9].

Sex-specific injury burden

Males had a greater level of injury than females (see Figure 5). In 2023, the male-to-female ratio was 1.03 for ASIR, 1.54 for ASDR and 1.51 for ASDAR, which shows that males had considerably higher rates of injury-related mortality and disability than females. During the study period, ASRs decreased in both men and women.

**Figure 5:**
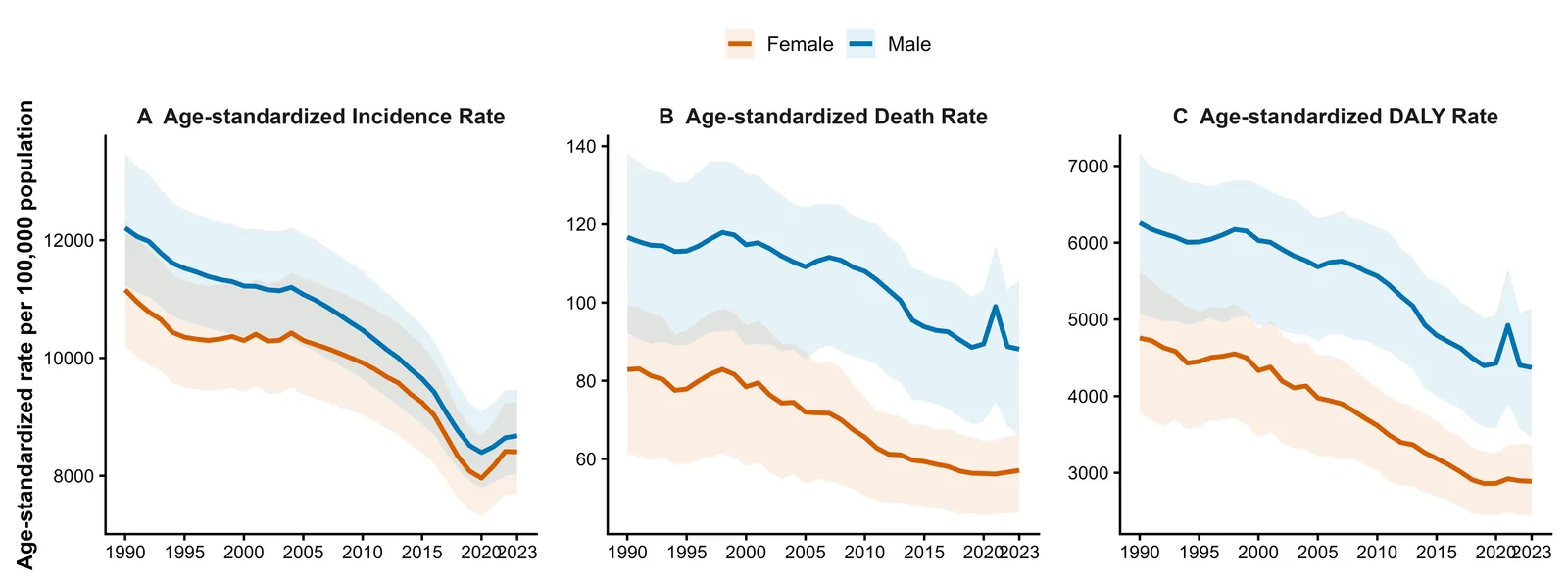
National trends in (A) age-standardized incidence rate (ASIR), (B) age-standardized death rate (ASDR), and (C) age-standardized disability-adjusted life year (DALY) rate (ASDAR) for injuries among males and females in India, 1990-2023. Age-standardized rates are expressed per 100,000 population. Estimates were obtained from the Global Burden of Disease (GBD) Study 2023 [9]. Solid lines represent point estimates, and shaded bands indicate 95% uncertainty intervals.

Age-specific injury burden

When analysed according to age, there were different patterns throughout the course of life (see Figure 6). The rate of injury was highest among people aged 20 to 24 years, fell during middle adulthood, and then rose in the elderly age groups. On the other hand, the rates of DALYs and mortality increased as age increased, the highest rates being found among those aged 85 years and over.

**Figure 6:**
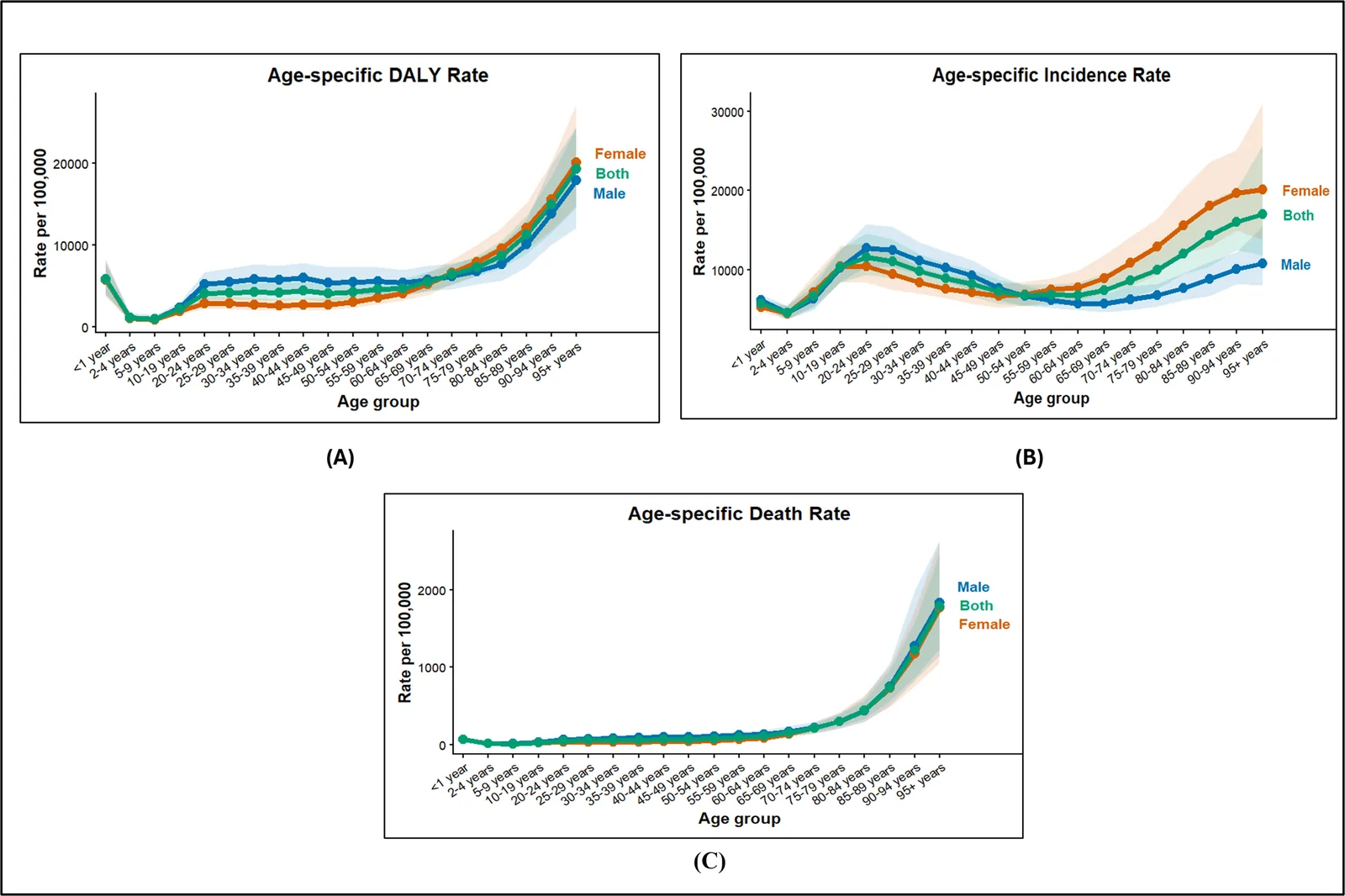
Age-specific (A) disability-adjusted life year (DALY), (B) incidence and (C) death rates for injuries in India by sex, 2023. Age-specific rates are expressed per 100,000 population. Estimates were obtained from the Global Burden of Disease (GBD) Study 2023 [9]. Solid lines represent point estimates, and shaded bands indicate 95% uncertainty intervals.

Joinpoint regression analysis

The joinpoint regression analysis found three statistically significant joinpoints for each of the age-standardized measures from 1990 through 2023 (as shown in Table 3 and Figure 7). The AAPC was -0.92% (95% CI: -1.00 to -0.84) for ASIR, -0.67% (95% CI: -0.74 to -0.59) for ASPR, -0.89% (95% CI: -1.02 to -0.74) for ASDR, and -1.15% (95% CI: -1.25 to -1.03) for ASDAR (in all cases P<0.001), which shows that there were statistically significant decreases in all of the age-standardized measures during the study period.

**Table 3:** Joinpoint regression analysis of age-standardized injury burden in India, 1990-2023. Age-standardized rates are expressed per 100,000 population. Estimates were obtained from the Global Burden of Disease (GBD) Study 2023 [9]. Abbreviations: AAPC: Average Annual Percent Change; ASDAR: Age-standardized Disability-Adjusted Life Year Rate; ASDR: Age-standardized Death Rate; ASIR: Age-standardized Incidence Rate; ASPR: Age-standardized Prevalence Rate.

Measure	No. of Joinpoints	AAPC (%)	95% CI	P value	Overall trend
ASIR	3	-0.92	-1.00 to -0.84	<0.001	Significant decrease
ASPR	3	-0.67	-0.74 to -0.59	<0.001	Significant decrease
ASDAR	3	-1.15	-1.25 to -1.03	<0.001	Significant decrease
ASDR	3	-0.89	-1.02 to -0.74	<0.001	Significant decrease

**Figure 7:**
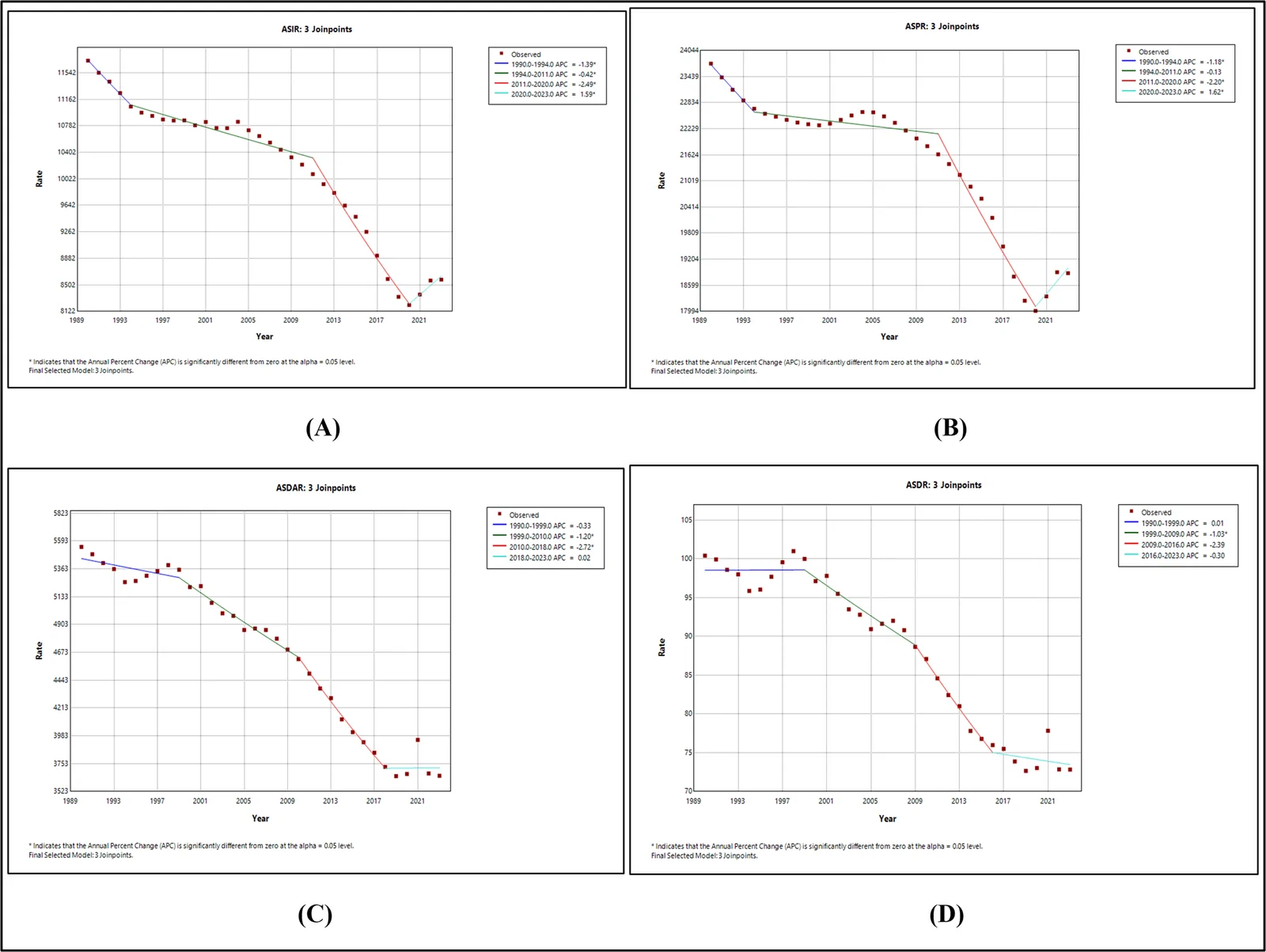
Joinpoint regression analysis of age-standardized incidence (A), prevalence (B), disability-adjusted life year (DALY) (C) and death (D) rates due to injuries in India, 1990-2023. Age-standardized rates are expressed per 100,000 population. Estimates were obtained from the Global Burden of Disease (GBD) Study 2023 [9].

## Discussion

The study provides a thorough examination of how injury patterns in India have changed over time, across regions and among different sexes and age groups, based on data from the GBD 2023 study. The findings reveal that the total number of injury incidence, deaths, and DALYs increased during this period but the ASRs decreased. Between 1990 and 2023, injury incidence rose by 25.1%, prevalence by 66.6%, deaths by 44.4%, and DALYs by 18.3%. At the same time, the ASIR, ASPR, ASDR, and ASDAR all fell by 26.8%, 20.5%, 27.5%, and 34.1%, respectively. This is important since the absolute numbers are influenced by population growth, demographic changes, and changes in risk, whereas the age-standardized rates give a better indication of how injury risk within the population has changed over time. The same pattern-increasing absolute numbers but decreasing age-standardized rates-has been observed in global studies of injuries and other major health problems, demonstrating the effect of growing population and ageing on the burden of disease [5,7].

The decline in age-standardized injury rates suggests an overall reduction in injury burden after accounting for changes in population age structure. The overall EAPC was -0.98% for ASIR, -0.71% for ASPR, -1.11% for ASDR and -1.38% for ASDAR, which shows steady improvement in all four of the main indicators. The Joinpoint analysis also revealed clear overall declines, with AAPCs of -0.92% for ASIR, -0.67% for ASPR, -0.89% for ASDR, and -1.15% for ASDAR. The study showed that the mortality and health loss due to injury have fallen a bit faster than the number of new or continuing cases. The faster decline in mortality and DALY rates may reflect improvements in trauma care, emergency services, rehabilitation, and broader health-system capacity; however, these potential explanations cannot be established from the present ecological analysis and should be interpreted as hypotheses rather than causal explanations. The GBD framework makes use of data from a number of different sources and applies statistical models to deal with any gaps or differences in the data, enabling comparisons of trends over time and across regions [5,10].

An important finding is the variation in injury levels across India's states and union territories. In 2023, Telangana, Uttarakhand, and Chhattisgarh had among the highest ASDARs, whereas Delhi, Mizoram, and Kerala had among the lowest. The burden-trend quadrant analysis revealed that a high current level of burden does not necessarily indicate that improvements are happening quickly. Telangana, Uttarakhand, Chhattisgarh, Haryana, and Odisha had high burdens together with slow progress, while Tamil Nadu, Karnataka, Madhya Pradesh, and Andhra Pradesh also had high burdens but were seeing faster improvement. States classified as having high burden and slow improvement may warrant further investigation and prioritization for injury-prevention efforts. However, the quadrant analysis is descriptive and does not establish the causes of state-level differences or identify which interventions would be most effective [5,7].

When the analysis was carried out by sex it was found that men still have a greater injury burden than women. In 2023, the male-to-female ratio was approximately 1.03 for ASIR, 1.54 for ASDR, and 1.51 for ASDAR. This indicates that, after allowing for age, men and women have similar rates of injury, but men have higher rates of death and disability due to injury. It therefore appears that sex differences are more important with regard to serious and fatal injuries than when it comes to the frequency of injuries occurring. Factors such as job hazards, road traffic, risk-taking, and violence may be involved, although this study cannot establish the exact causes. This finding is in agreement with other GBD results which show that men have a higher injury burden in many places [7].

The findings for different age groups show the importance of considering all age groups when planning injury prevention. Although young adults, in particular those between 20 and 24 years of age, had the highest injury incidence rates, the rates of death and DALYs increased with age and were highest among people who were 85 years old and older. Consequently, the groups that experience the most injuries are not necessarily the ones that suffer the most serious health consequences. It is true that younger people tend to have more injuries, but older adults are more likely to suffer serious harm or to die as a result of injuries. For health policy this means that prevention efforts should not only concentrate on young people or on injuries relating to roads and at work, but also take into account the growing risks faced by older adults.

The results have several consequences for public health policy in India. Firstly, since the total number of injuries is still rising, a falling ASR must not be taken to mean that the injury problem has been eliminated; a growing and ageing population can increase the number of people who need injury prevention, emergency care and rehabilitation. Second, the large differences between states show that injury prevention measures should be aimed at specific geographical areas rather than being applied in the same way throughout the country. Third, those states which have a heavy burden and are showing little improvement should be given priority for extra prevention efforts and for strengthening of the health system. Finally, the fact that mortality and DALYs are so heavily concentrated among men and older people suggests that prevention programmes should include approaches that are tailored to the particular population groups. These findings justify using routinely updated disease burden estimates to identify the populations and geographical areas that need more attention [5,10].

Strengths

One of the advantages is that it makes use of the GBD 2023 estimates, allowing for a consistent evaluation of injury incidence, prevalence, mortality, and DALYs in India and its states and union territories over the 34-year-period from 1990 to 2023. The study combined age-standardized rates with absolute figures and included an analysis that was specific to sex, age, and geography. The application of EAPC and Joinpoint regression offered two complementary methods for examining long-term and changing temporal trends, and the burden-trend quadrant analysis was used to identify the states that need more attention from public health services [5,8,12,13]. Moreover, the employment of the standardized GBD methodology and the GATHER reporting guidelines further ensures that the results are comparable and reproducible [5,8,10].

Limitations

There are certain limitations to the study. Since it is an ecological analysis relying on modelled GBD estimates, the results are affected by the limitations relating to the availability, quality and completeness of the underlying data and therefore cannot be regarded as direct measurements [5,10]. It is not possible for the study to establish individual-level causal relationships between the burden of injury and potential determinants such as socioeconomic conditions, healthcare access or infrastructure. The state-level administrative groupings in the GBD database might also differ from the present boundaries. Moreover, the analysis looked at the overall burden of injury rather than at the specific mechanisms of injury, which limits the interpretation of cause-specific patterns. Consequently, prevention recommendations based on the aggregate burden should be interpreted cautiously and should not be considered substitutes for cause-specific injury analyses. Future studies should examine individual injury causes and their associated risk factors to inform more targeted prevention strategies. Furthermore, temporal dependence was not explicitly modelled in the present analysis because a year-specific variance-covariance matrix was not available; therefore, the p-values and confidence intervals from the Joinpoint regression should be interpreted with caution. The burden-trend quadrant analysis was also a descriptive prioritization approach based on the median age-standardized DALY rate in 2023 and median EAPC for 1990-2023. The categories of “rapid” and “slow” improvement are relative to the observed distribution of states and union territories and should not be interpreted as absolute, validated epidemiological or policy-performance thresholds. While the burden-trend analysis identifies the priority states it cannot say which specific interventions led to the observed improvements.

## Conclusions

Over the past three decades, India has experienced a substantial decline in the age-standardized burden of all injuries, although the absolute number of injuries, deaths, and DALYs remains considerable. Marked variation across states, age groups, and sex indicates that the injury burden is not uniformly distributed and remains an important public health concern. However, these findings are descriptive and should not be interpreted as establishing causal explanations for the observed trends or differences.

The findings may help inform injury-prevention priorities and subnational surveillance, particularly in states and population groups with comparatively higher burdens. However, the burden-trend quadrant analysis represents a descriptive prioritization approach and does not establish which specific interventions are responsible for observed improvements or which interventions would be most effective. Further cause-specific and intervention-focused studies are needed to guide targeted injury-prevention strategies and support evidence-based allocation of resources.

## Appendices

**Table 4:** Global Burden of Disease (GBD) 2023 Results Tool extraction parameters used for the analysis of injury burden in India, 1990-2023. The analysis used the aggregate GBD “Injuries” cause category and did not separately analyze individual injury causes. Age-standardized estimates were used for national and state-level comparisons, whereas age-specific estimates were used to describe variation across age groups. All estimates were obtained from the GBD 2023 Results Tool [9] and are reported with corresponding 95% uncertainty intervals where applicable.

Analysis	Parameter	GBD Results Tool selection
National and State level trends of injuries	GBD Estimate	Cause of Death or injury
	Measure	Incidence, Prevalence, Death, DALYs (Disability-Adjusted Life Years)
	Metric	Number, Rate
	Causes	Injuries (aggregate)
	Location	India and 31 GBD geographical units representing Indian states and union territories
	Age	All ages, Age -standardized
	Sex	Both
	Year	1990 to 2023
Sex-specific injury burden	GBD Estimate	Cause of Death or injury
	Measure	Incidence, Death, DALYs (Disability-Adjusted Life Years)
	Metric	Rate
	Causes	Injuries (aggregate)
	Location	India
	Age	All ages, Age -standardized
	Sex	Male, Female
	Year	1990 to 2023
Age-specific injury burden	GBD Estimate	Cause of Death or injury
	Measure	Incidence, Death, DALYs (Disability-Adjusted Life Years)
	Metric	Rate
	Causes	Injuries (aggregate)
	Location	India
	Age	<1 year, 2-4 years, 5-9 years, 10-19 years, 20-24 years, 25-29 years, 30-34 years, 35-39 years, 40-44 years, 45-49 years 50-54 years, 55-59 years, 60-64 years, 65-69 years, 70-74 years, 75-79 years, 80-84 years, 85-89 years, 90-94 years, 95+ years
	Sex	Male, Female, Both
	Year	1990 to 2023
All Analyses	Data extraction date	27-07-2026
